# Outcome of laparotomy and conservative treatment of patients with acute mesenteric venous ischemia with viable bowel

**DOI:** 10.1007/s00068-022-01924-z

**Published:** 2022-03-09

**Authors:** Sameh Hany Emile, Ahmed Magdy Elmetwally, Ahmed AbdelMawla

**Affiliations:** 1 Colorectal Surgery Unit, General Surgery Department, Mansoura University Hospitals, Mansoura University, Mansoura, Egypt; 2 Vascular Surgery Department, Mansoura University Hospitals, Mansoura University, Mansoura, Egypt

**Keywords:** Acute mesenteric ischemia, Non-therapeutic, Laparotomy, Impact, Outcome

## Abstract

**Background:**

Acute mesenteric ischemia (AMI) is one of the most serious abdominal emergencies. Predicting the onset of bowel necrosis that warrants surgical intervention is of paramount importance in AMI. The present study aimed to investigate the outcome of patients with AMI secondary to mesenteric venous occlusion (MVO) and the consequence of non-therapeutic exploratory laparotomy.

**Methods:**

The records of 132 patients with AMI were retrospectively reviewed. The outcome of patients with acute mesenteric venous ischemia (AMVI) and viable bowel was analyzed based on the method of treatment: conservative versus surgical. The impact of non-therapeutic laparotomy on the outcome of patients with AMVI in terms of morbidity, readmission, and mortality was analyzed.

**Results:**

Forty-seven patients (34 male) with AMVI had viable bowel. Of the 47 patients with viable bowel, 8 (17%) had an exploratory non-therapeutic laparotomy, whereas 39 patients were treated conservatively. Patients who had non-therapeutic laparotomy had significantly higher complication (50 vs 5.1%, *p* = 0.005) and readmission rates (37.5 vs 5.1%, *p* = 0.03) and longer hospital stay (8.5 vs 7 days, *p* = 0.02) than those treated conservatively. Patients with bowel necrosis who had a therapeutic laparotomy had slightly lower rates of morbidity and mortality as compared to patients with viable bowel who underwent a non-therapeutic laparotomy.

**Conclusion:**

Careful assessment and informed decision-making in patients with AMVI are crucial to avoid unnecessary surgical intervention that can result in higher rates of complications and readmission and extended hospital stay.

**Supplementary Information:**

The online version contains supplementary material available at 10.1007/s00068-022-01924-z.

## Introduction

Acute mesenteric ischemia (AMI) is one of the most serious abdominal emergencies that, despite of its low incidence that accounts for < 0.2% of acute surgical admissions, can result in high mortality if left untreated [1]. Owing to the progressive nature of the disease, early diagnosis and prompt intervention are imperative to decrease the incidence of bowel necrosis and subsequent morbidities.

AMI can be broadly classified into occlusive and non-occlusive ischemia. The mesenteric occlusive ischemia is either due to occlusion of the mesenteric arteries by embolism or thrombosis or occlusion of the mesenteric veins by venous thrombosis. On the other hand, non-occlusive mesenteric ischemia (NOMI) involves persistent spasm of the mesenteric vessels due to severe hypotension or induced by drugs such as vasopressors, ergotamine, and digitalis [2, 3].

Establishing the diagnosis of AMI and determining whether bowel necrosis has occurred or not requires comprehensive assessment. Although there are no specific laboratory parameters to confirm the diagnosis of AMI, some parameters such as the mean platelet volume, Neutrophil to Lymphocyte Ratio (NLR), red cell distribution width (RDW), lactate, and D-dimer maybe helpful to suggest the diagnosis [4]. Contrast CT scanning is a valuable tool for the assessment of mesenteric ischemia with a specificity exceeding 95% [5].

The most serious consequence of AMI is the development of bowel necrosis. Failure to recognize non-viable intestine in a timely manner can result in multi-system organ dysfunction and high incidence of mortality. Prompt laparotomy allows for direct assessment of bowel viability, yet can be associated with a number of adverse effects. Hence, the management of AMI requires achieving a fine balance between the need for urgent surgical intervention in case bowel necrosis has supervened, and the need to avoid unnecessary laparotomy in the case of bowel ischemia without evident bowel necrosis or infarction.

The present study aimed to investigate the outcome of patients with AMI secondary to mesenteric venous occlusion (MVO) who had viable bowel and the consequence of non-therapeutic exploratory laparotomy.

## Patients and methods

### Study design and setting

This was a retrospective cohort study of patients with acute mesenteric venous ischemia (AMVI). The study took place in the Emergency Department and the General Surgery Department of Mansoura University hospital in the period of January 2011 through July 2020. Ethical approval was obtained from the institutional review board of Mansoura University, Faculty of medicine (R.20.08.996).

### Eligibility criteria

Inclusion criteria were: consecutive adult patients of either sex with AMI whether were treated conservatively or with exploratory laparotomy. The main focus of the study was patients with AMVI who had viable bowel. Patients with AMI who had bowel necrosis and underwent therapeutic laparotomy and bowel resection were included to the study for comparison with patients who had non-therapeutic laparotomy.

Exclusion criteria were: patients with diagnoses other than AMI and patients with incomplete records missing important data on the outcomes.

### Assessments

Patients with AMI were assessed by a comprehensive assessment plan that entailed detailed history taking, clinical examination, and investigations. Patients were queried about the onset and duration of their complaint, associated abdominal symptoms, upper or lower GI bleeding, medical comorbidities, and history of previous surgeries. General examination, including vital sign measurement, was followed by abdominal examination to reveal signs of peritonitis including abdominal tenderness, rigidity, or abdominal masses.

Routine laboratory investigations were ordered, including complete blood count, SGOT/SGPT, serum albumin, serum creatinine, coagulation profile, and random blood glucose. When AMI was suspected, serum amylase and lactate levels were ordered. A suspected initial diagnosis of AMI was made based on clinical and laboratory parameters and was confirmed with abdominal CT scanning with intravenous contrast that showed signs of AMI. This was in line with the guideline devised by the World Society of Emergency Surgery (WSES) [1].

### Management strategy

Upon resuscitation of the patients, a decision on management was made based on the outcome of the comprehensive assessment plan. Patients with AMVI were treated either conservatively or by exploratory laparotomy. The management strategy was in compliance with the WSES guideline [1].

#### Conservative treatment

Conservative treatment included fluid resuscitation with IV crystalloids to enhance visceral perfusion, correction of electrolyte abnormalities, and insertion of nasogastric tube to decompress the stomach and bowel. Intravenous broad-spectrum antibiotics (1 gm of cefotaxime/12 h and 500 mg of metronidazole/8 h) were started and low-molecular weight heparin (enoxaparin 80 IU) was given subcutaneously. Patients were monitored closely with measurement of vital signs every 6 h and complete blood count done every 12 h. If signs of peritonitis developed, leukocyte count showed marked rise, or hemodynamic instability supervened, the patient was shifted to exploratory laparotomy.

#### Surgical treatment

Indications for surgical intervention included patients with persistent signs of overt peritonitis, patients with hemodynamic instability not responding to adequate resuscitation, and patients with suggestive signs of bowel necrosis in CT scanning such as pneumatosis intestinalis, dilated bowel loops, and free intraperitoneal fluid [6].

Surgical treatment entailed a midline exploratory laparotomy with careful assessment of all areas of bowel for viability. Patients with evident bowel necrosis underwent resection of the necrotic bowel segment and primary end-to-end anastomosis (therapeutic laparotomy) while in patients with ischemic, yet viable bowel the bowel viability was confirmed then the bowel was returned to the peritoneal cavity and the abdomen was closed (non-therapeutic laparotomy).

**Fig. 1:**
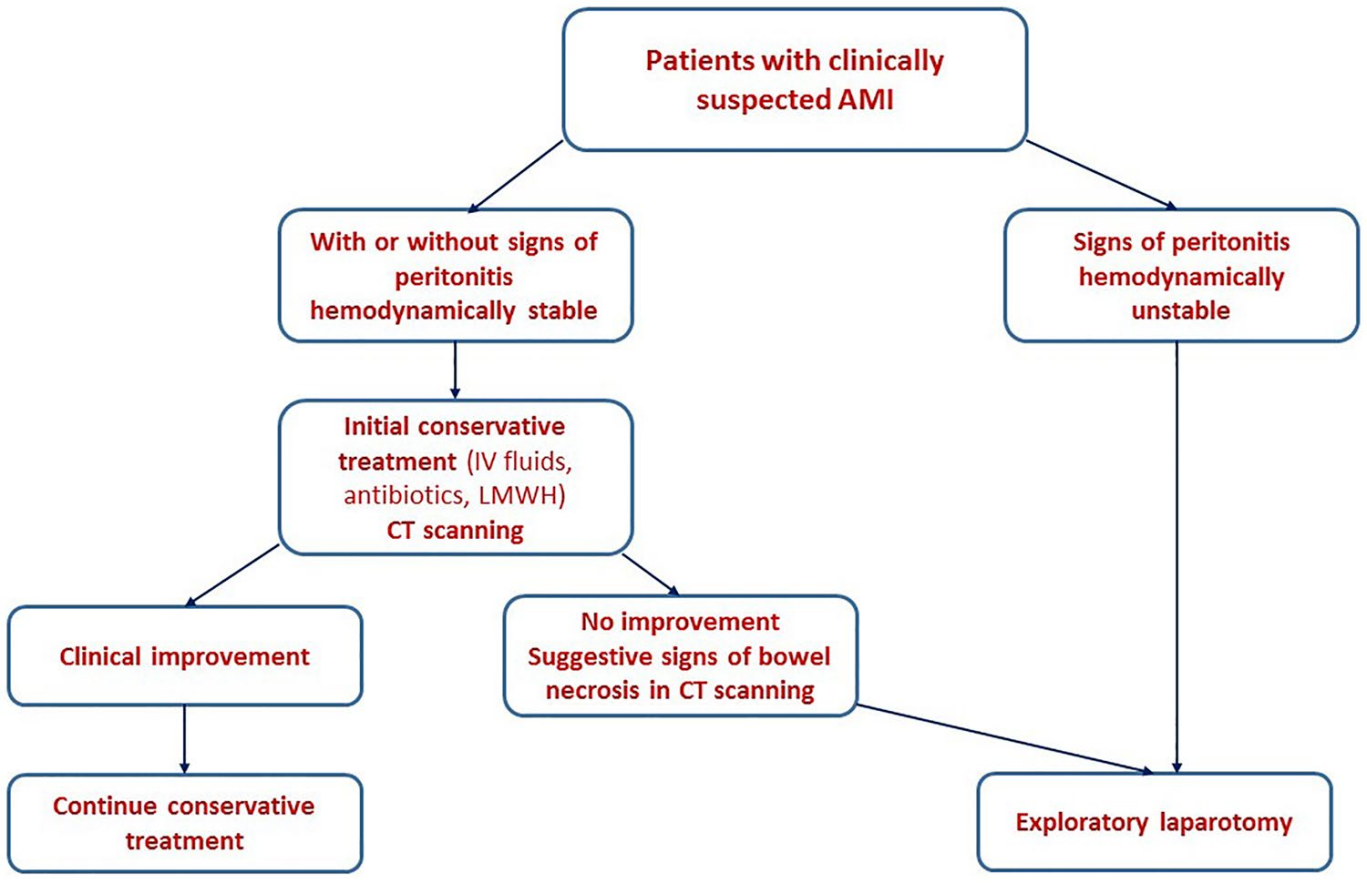
Algorithm used for decision making

Since the study included only patients with AMI secondary to mesenteric venous thrombosis, there was no place for vascular repair or thrombectomy. The algorithm used for decision making in patients with AMI is shown in Fig. 1.

### Data collection

Data collected included the characteristics of patients with AMVI including age and sex, comorbidities, vital signs, leukocyte and platelet count, arterial pH, CT findings, type and outcome of treatment in terms of complications (surgical site infection or occurrence, intra-abdominal collection or abscess, bowel injury, leakage, hematemesis, and medical complications), mortality, and readmission rates. Complications were graded according to the Clavien–Dindo classification.

### Sample size and statistical analysis

Since the study was a retrospective review of data, a convenient sample size of all eligible patients available was selected.

Statistical analysis was done using SPSS version 23™ (IBM Corp; Chicago, USA). Continuous variables were expressed as mean and standard deviation (SD) or median and range and categorical variables as numbers and proportions. Student *t* test or Mann–Whitney test was used to process continuous data and Fisher exact test or Chi-square test was used to process categorical data. *P* values < 0.05 were considered significant.

## Results

### Patients’ characteristics

**Fig. 2:**
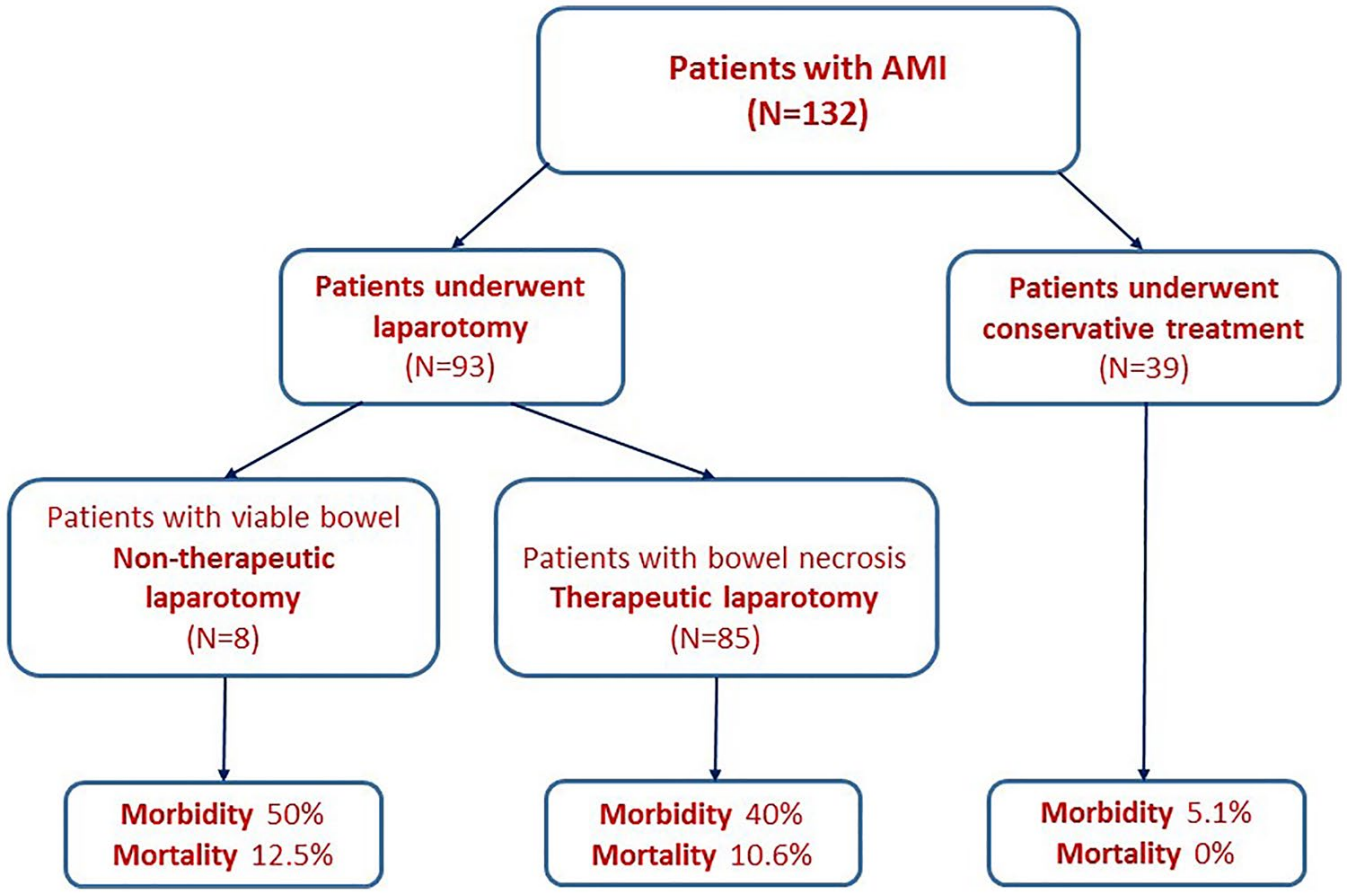
Flow chart of patient selection to the study

During the study period, 132 patients with AMI were admitted to the Emergency Department, of whom 85 (64.4%) had bowel necrosis and underwent exploratory laparotomy with resection of the necrotic bowel. The remaining 47 (35.6%) patients had viable bowel (Fig. 2). Seven patients had superior mesenteric venous thrombosis, another 7 had portal vein thrombosis, while combined portal and superior mesenteric vein thrombosis was detected in 33. Most of these cases were secondary to portal hypertension.

Patients were 34 (72.3%) men and 13 (27.7%) women of a median age of 55 (range, 27–77) years. Eight (17%) patients had DM, 12 (25.5%) had hypertension, 20 (42.5%) had ischemic heart disease, and 6 (12.7%) had chronic liver disease. Twenty (42.5%) patients had signs of peritonitis. Regarding vital signs on admission, the mean body temperature was 37.1 ± 0.47 C, the mean pulse rate was 90.5 ± 12.4 bpm, and the mean systolic blood pressure was 117.4 ± 12.9 mmHg. The mean total leukocyte count was 13 ± 4.6, mean platelet count was 232.9 ± 88.7, and mean arterial pH was 7.41 ± 0.04.

Of the 47 patients with viable bowel, 8 (17%) were treated with exploratory laparotomy that revealed ischemic bowel loops that did not warrant resection. The other 39 patients were treated conservatively and showed remarkable clinical improvement and were discharged without surgical intervention.

The baseline characteristics of patients with viable bowel who had or did not have laparotomy were similar with no significant difference in regards to age, sex, comorbidities, vital signs, and laboratory parameters. More patients who underwent non-therapeutic laparotomy had signs of peritonitis, suggestive CT signs of bowel necrosis, and higher total leukocyte count than patients who were treated conservatively (Table 1).

**Table 1:** Baseline characteristics of patients with AMI and viable bowel who had or did not have non-therapeutic laparotomy

Variable	Non-therapeutic laparotomy	Conservative treatment	*P* value
Number	8	39	–
Mean age in year	53.9 ± 17.3	54.8 ± 12.9	0.87
Male/female	6/2	28/11	0.99
Comorbidities			
Diabetes mellitus	2 (25)	6 (15.3)	0.61
Hypertension	1 (12.5)	11 (28.2)	0.66
Ischemic heart disease	3 (37.5)	17 (43.5)	0.99
Chronic liver disease	2 (25)	4 (10.2)	0.27
Signs of peritonitis (%)	8 (100)	12 (30.7)	**0.0004**
Mean body temperature (C)	37 ± 0.4	37.1 ± 0.6	0.96
Mean pulse rate/minute	84.5 ± 3.6	91.7 ± 17	0.28
Mean systolic blood pressure in mmHg	115 ± 11.9	117.9 ± 13.2	0.3
Mean total leukocyte count	15.4 ± 2.8	12.5 ± 6.2	**0.008**
Mean platelet count	255.4 ± 55.6	228.4 ± 27.8	**0.007**
Mean arterial pH	7.44 ± 0.04	7.4 ± 0.03	0.4
CT findings suggestive of bowel necrosis	4 (50)	3 (7.7)	**0.01**

Bold values indicate significant *p* values less than 0.05

### Patients’ outcome

**Fig. 3:**
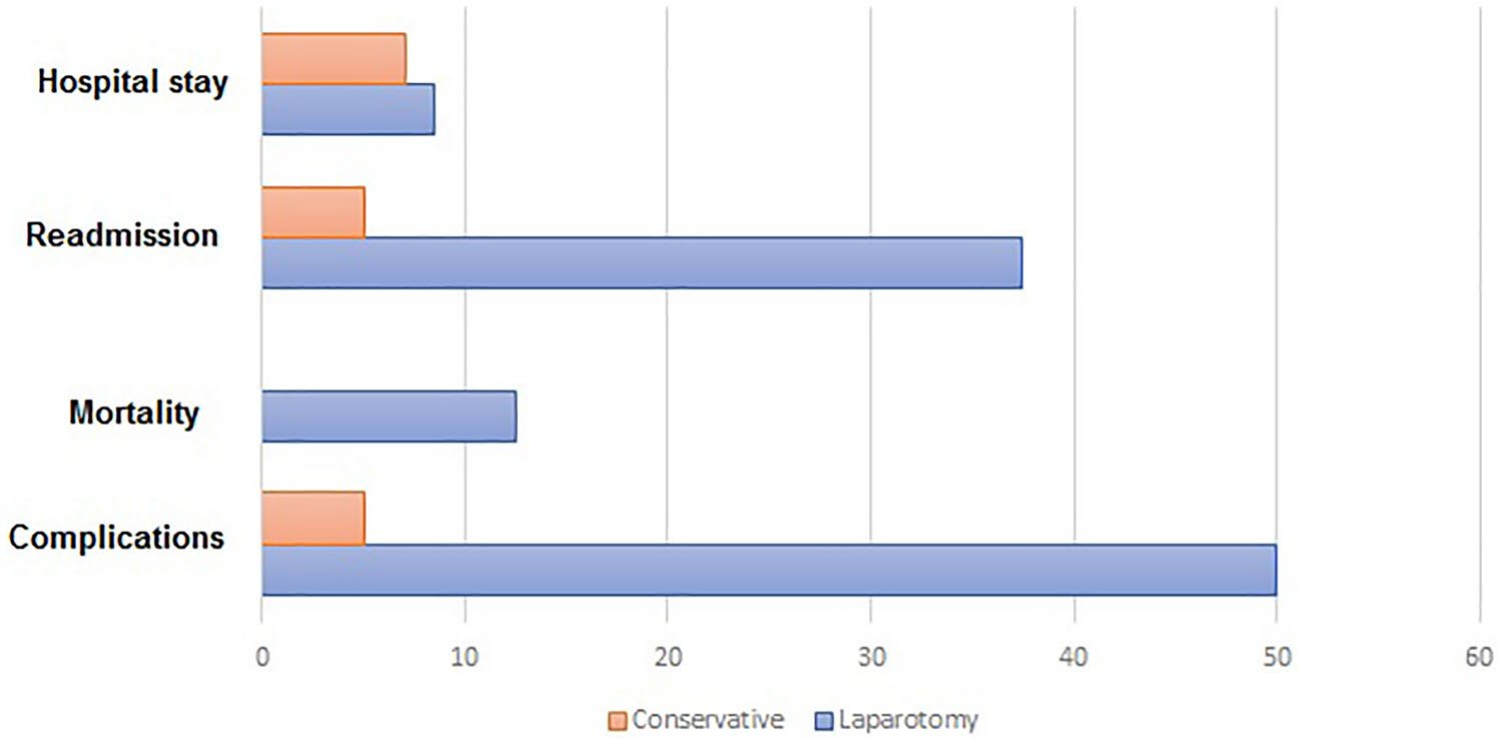
Outcome of patients with viable bowel who had non-therapeutic laparotomy or conservative treatment

Patients with viable bowel who had non-therapeutic laparotomy had significantly higher complication rate (50 vs 5.1%, *p* = 0.005), higher readmission rate (37.5 vs 5.1%, *p* = 0.03), and longer hospital stay (8.5 vs 7 days, *p* = 0.02) than those treated conservatively (Table 2) (Fig. 3).

**Table 2:** Outcome of patients with AMVI and viable bowel who had or did not have non-therapeutic laparotomy

Variable	Non-therapeutic laparotomy (*n* = 8)	Conservative treatment (*n* = 39)	*P* value
Median hospital stay (range)	8.5 (7–10)	7 (6–9)	**0.02**
Complications (%)	4 (50)	2 (5.1)	**0.005**
Type of complications	Surgical site infection (*n* = 1) Decompensation of chronic liver disease (*n* = 2) Wound collection and dehiscence (n = 1)	Decompensation of chronic liver disease (*n* = 1) Hematemesis (*n* = 1)	–
30-day readmission (%)	3 (37.5)	2 (5.1)	**0.03**
Mortality (%)	1 (12.5)	0	0.17
Clavien-Dindo classification of morbidities			
I	1 (12.5)	0	0.39
II	2 (50)	1 (2.6)	
III	0	1 (2.6)	
IV	1 (12.5)	0	
V	1 (12.5)	0	

Bold values indicate significant *p* values less than 0.05

There was no difference between the two groups in overall mortality (12.5% vs 0, *p* = 0.17) and grade of complications (*p* = 0.39). Complications that developed in the non-therapeutic laparotomy group entailed surgical site infection (*n *= 1), decompensation of chronic liver disease (*n* = 2), and wound collection and dehiscence (*n* = 1). The conservative treatment group experienced two complications: decompensation of chronic liver disease and hematemesis.

### Comparing patients’ characteristics and outcomes in the therapeutic laparotomy, non-therapeutic laparotomy, and conservative management groups

Patients who had a therapeutic laparotomy (resection of necrotic bowel) had higher mean pulse rate, lower mean systolic blood pressure, higher mean total leukocyte count, and more suggestive signs of bowel necrosis in CT scanning than did patients in the non-therapeutic laparotomy and conservative groups. The three groups were similar in terms of age, sex distribution, comorbidities, mean body temperature, and arterial PH. The complication and mortality rates in the non-therapeutic laparotomy group were slightly higher than those in the therapeutic laparotomy group, yet much higher than those in the conservative treatment group (Table 3).

**Table 3:** Patients’ characteristics and outcomes in the therapeutic laparotomy, non-therapeutic laparotomy, and conservative management groups

Variable	Therapeutic laparotomy	Non-therapeutic laparotomy	Conservative treatment
Number	85	8	39
Mean age in year	55.6 ± 11	53.9 ± 17.3	54.8 ± 12.9
Male (%)	60 (70.4)	6 (75)	28 (71.8)
Diabetes mellitus	17 (20)	2 (25)	6 (15.3)
Hypertension	10 (11.8)	1 (12.5)	11 (28.2)
Ischemic heart disease	33 (38.8)	3 (37.5)	17 (43.5)
Chronic liver disease	21 (24.7)	2 (25)	4 (10.2)
Signs of peritonitis (%)	85 (100)	8 (100)	12 (30.7)
Mean body temperature (C)	37.4 ± 13.1	37 ± 0.4	37.1 ± 0.6
Mean pulse rate/minute	95.2 ± 36.2	84.5 ± 3.6	91.7 ± 17
Mean systolic blood pressure in mmHg	107.8 ± 38.7	115 ± 11.9	117.9 ± 13.2
Mean total leukocyte count	20.4 ± 8.2	15.4 ± 2.8	12.5 ± 6.2
Mean platelet count	220.7 ± 122.1	255.4 ± 55.6	228.4 ± 27.8
Mean arterial pH	7.4 ± 2.6	7.44 ± 0.04	7.4 ± 0.03
CT findings suggestive of bowel necrosis	30/36 (83.3)	4/8 (50)	3/39 (7.7)
Complications (%)	34 (40)	4 (50)	2 (5.1)
Mortality (%)	9 (10.6)	1 (12.5)	0

### Diagnostic accuracy of the algorithm followed for decision making

The algorithm followed for decision making in the present study had an overall sensitivity of 100% (95% CI: 95.7–100), specificity of 82.9 (95% CI: 96.2–92.3), and accuracy of 93.9% (95% CI: 88.4–97.3). The positive predictive value was 91.4% and the negative predictive value was 100%.

## Discussion

Treatment of AMI is a challenging task and making an informed decision on AMI patients is crucial as faulty decisions may result in delaying surgery in patients with definite bowel necrosis with subsequent sequel including multiorgan dysfunction and death. On the other hand, a decision to do laparotomy in AMI patients with ischemic, yet viable bowel is not only considered an unnecessary intervention but can also be associated with added morbidity, extended stay, and higher health care costs [2].

To make an informed decision on which treatment strategy is best for every AMI patient, one should predict bowel necrosis warranting surgical intervention beforehand. To this end, different studies [7–9] investigated the predictors of bowel necrosis in AMI that justify the need for surgery. Several predictive factors for bowel necrosis were reported in the literature and a recent meta-analysis [10] compiled these factors into a comprehensive prognostic scoring system.

While the previous studies [4, 10] were focused upon the diagnostic parameters of AMI and the risk factors of bowel necrosis, no emphasis was made on the impact of non-therapeutic laparotomy on the outcome of patients with AMI and viable bowel. Thus, we conducted a review of prospective data of patients with AMVI who had viable bowel to determine the impact of non-therapeutic laparotomy on their outcome as compared to patients who had conservative treatment. We chose patients with AMI secondary to mesenteric venous thrombosis because surgical intervention in these patients aims to resect the necrotic bowel only with no place for vascular repair, thrombectomy, or embolectomy, unlike with acute mesenteric arterial ischemia in which case laparotomy maybe otherwise useful and therapeutic.

Although AMVI represents around 10% of AMI cases as reported in the literature [2, 3], this condition is more common in Egypt because of the prevalence of hepatitis C virus in the country [11], with its secondary effects including liver cirrhosis and portal hypertension. Moreover, the prevalence of schistosomiasis that is also associated with portal hypertension can factor in the higher incidence of AMVI. Portal hypertension is associated with considerable venous congestion in the mesenteric circulation with portomesenteric venous thrombosis, which renders the patients more susceptible to AMVI [12].

Approximately, two-thirds of patients with AMI had evident bowel necrosis justifying surgical intervention, whereas only one-third had viable bowel. This was in discordance to the incidence of irreversible bowel necrosis in AMI in the literature (42%) and could be attributed to the delayed presentation of the patients and poor function of the venous collaterals that failed to relieve the venous congestion [10].

Among 47 patients with AMI and viable bowel, 17% were elected to receive an exploratory laparotomy based on the presence of signs of overt peritonitis or suggestive signs of bowel necrosis in CT scanning. This high false-negative rate attests for the challenges encountered by the clinicians when making a decision on AMI [13]. Patients with viable bowel who had exploratory laparotomy had obvious signs of peritonitis and suggestive signs of bowel necrosis in CT scanning; however, laparotomy was not necessary in these patients because they did not have evidence of bowel necrosis. This observation raises a question on the diagnostic accuracy of these parameters in the setting of AMI.

It was obvious that non-therapeutic laparotomy had a negative impact on the outcome of AMVI patients. It was associated with longer stay and higher complication and readmission rates as compared to AMVI patients who were treated conservatively. Half of the complications in the laparotomy group were related to the surgical incision itself, in the form of infection or wound dehiscence. The two patients with chronic liver disease in the laparotomy group developed decompensation of hepatic disease and coma versus only one out of four patients in the conservative treatment group. This highlights the negative impact of surgery and anesthesia on patients with chronic liver disease who should be treated conservatively whenever possible [14].

The extended hospital stay after non-therapeutic laparotomy may be explained by the extra time needed for recovery after surgery, especially in patients with medical comorbidities which has been previously demonstrated by another study on non-operative treatment of AMVI [15]. The incidence of hospital readmission was higher after laparotomy than after conservative treatment and most of these readmissions were related to the adverse effects of surgery.

The present study has a number of limitations being a single-center experience entailing small numbers of patients. In addition, the retrospective nature of the study can be associated with an inherent risk of bias. The impact of non-therapeutic laparotomy on other parameters such as return to work and daily activities, resumption of normal oral intake, and quality of life was not investigated which warrants further prospective trials.

## Conclusion

Non-therapeutic laparotomy was associated with longer stay and higher complication and readmission rates as compared to AMVI patients who were treated conservatively. Therefore, avoiding unnecessary laparotomy in AMVI is of paramount importance to avoid these adverse effects.

### Supplementary Information

Below is the link to the electronic supplementary material.

Supplementary file1 (DOC 82 KB)
